# Informed consent: Are we doing enough?

**DOI:** 10.4103/2229-3485.71769

**Published:** 2010

**Authors:** Pranati

**Affiliations:** *Institute for One World Health, Liasion Field Office, Anand Vihar Building, 1st Floor, West Boring Canal Road, Patna-800001, Bihar, India*

**Keywords:** Autonomy, clinical trials, decision aids, informed consent, research ethics

## Abstract

The process of informed consent is an ethical mandate for all clinical trials. The principles of ethics in research involving human beings decrees the conduct of a process where the prospective research subjects should be informed of all aspects of the research study and after complete comprehension of all the features involved, should express willingness to be a part of the study, and the same should be duly documented. The consent should be fully informed and autonomous. However, with subject populations that are mostly medically naïve and for whom the whole concept of clinical research and the umpteen terms and concepts associated with it are alien; the true essence of an “informed” and “autonomous” decision is largely lost. The consent process thus gets reduced to mainly a “narration-followed-by-signature” process. Over the last few years, this gap in principles and practices of ethics and consent has been acknowledged and innovative concepts and attempts are being fostered, to make the informed consent process more “ethical”. The article discusses the loopholes associated with the informed consent process and the innovative concepts to make it more authentic.

The informed consent process and its adequate documentation are mandatory before any trial-related process may be administered over a potential research subject. All guidelines and laws pertaining to clinical trials pontificate the same ethical ideal; and it is the most sanctimonious ritual practiced in the industry. All trainings of investigators and research personnel strongly reiterate the principles of Belmont report and emphasize upon the requirements for the design of informed consent document and its adequacy in conduct. But are we actually doing enough?

Are signatures of subjects or representatives truly authoritative of the subjects’ complete understanding of all aspects of the study? Does an elaborate documentation of the process suffice in indicating that the subject has imparted a *pertinent informed and voluntary* consent for participation? For the educated, aware, health-savvy population this holds relevance. But in a country where nearly 35% population is illiterate, such an aware class of people forms a small sub group. The time and technicalities involved in the process may be intimidating to illiterate subjects whose awareness about medical and legal rights is low. Language, cultural and community barriers further increase the incompetence of consent process.[1] Moreover, how many people in our country are actually inquisitive about issues related to health and medicine? Isn’t the process of “informed consent” shrunken to simply a method of obtaining subject’s signatures on the document?[2]

Most of us go to doctors when ailing, and take the prescribed medication as indicated. Do we think about adverse effects of all the medicines pro-actively and ask our doctors about them? How many of us actually have turned the leaves of package inserts or read the scribbling over medicine strips? Do we understand the intricacies of medical procedures or even ask the medical practitioners what good or harm, or just the effect that they may cause to our body? It is a very small percentage of the billion-plus population that has the medically probing brain. We have a certain faith in the doctors that we approach for our sufferings and thus, whatever the physician advices is accepted to be the best therapeutic option for the benefit of the patient.[3] This fact of an implicit faith in the doctors kills the essence of an autonomous decision. As quoted very aptly in a study, “under such conditions, informed consent process becomes more of providing information to lead to a (therapist-determined) beneficial therapeutic outcome, rather than to enhance autonomous patient choice”.[4] How many times do we look up at the alternative medication available and have asked our doctors if the other one could be prescribed? *Do we pro-actively participate in our medical treatments?*

A fairly big subset of the population is educated and is expected to raise questions and seek clarifications at the mention of the fact that this is “research”. The word brings with it a sense of doubt and when in doubt the mind unfurls all censorious faculties. This is to talk of the educated class; but there is a mammoth subset of the semi-urban and rural population that is too medically naïve to appreciate the importance of an informed consent.[3,5] Does an informed consent document (ICD) suffice in ensuring the ‘*adequacy*’ of the process then?

Video-taping of the whole informed consent process and even a telephonic interview of random subjects by the ethics committee to gauge their understanding of the study are workable options. But how far can they be successful considering the nuances of subject confidentiality?

The loopholes in consent process have come to the fore over the last decade and researchers over the world are exploring innovative ways and means to strengthen the process in terms of patient comprehension and autonomy involved in it.[6]

The true context of the decision making process is often compromised by patient-centered barriers such as age, education and illness, and/or process-centered barriers like content and readability of form, timing of discussion, amount of time allotted for the process.[7] Moreover, the context of initial and continued comprehension of research participation is largely overlooked. Research has demonstrated that participants in randomized control trials often believe that they are receiving an active and proven drug or device.[8] and hence, may withdraw consent later upon realizing that their conditions are not improving (compared to other participants) and that they may not be getting the drug they had initially thought of. Such a scenario is most probably expected to result in embittered feelings and lead to consent withdrawals. Although, according to the rules of ethics all trial subjects are free to withdraw consent at any point in time without losing upon their optimal medical care; but if the withdrawals happen due to lack of information and understanding, then it becomes an issue that needs to be addressed more carefully. A truly informed and autonomous decision is not only an ethical obligation but will also ensure that participants who enter the study remain involved through the course of the study, thereby improving accrual, retention and scientific validity of the study.[9]

However, ensuring that consent is truly voluntary and fully informed remains challenging.

A composite approach including innovative processes of delivering the often complex information seems an answer, but limited action has been undertaken in this regard. All aspects of the process, such as the content of the informed consent form, methods of delivering the information to prospective subjects and ensuring their continued consent throughout the duration of study, need to be revisited.

The design and content of the consent form should be such that all information is understood and sinks within the prospective subjects so as to enable them to make an autonomous decision. It has been argued that complicated concepts conveyed in mostly lengthy and detailed consent forms, often for regulatory and institutional fulfillment, may in themselves pose the biggest barrier to the process.[10]

The task of conveying the concepts of research and basic scientific terms (blinding, randomization, confidentiality and others) to a scientifically naïve population is the major challenge of the informed consent process.[11] Considering the paucity in the local vocabulary for such terminology, the idea of achieving lower levels of literacy in drafting the consent form is also not workable.[11] It can be difficult to achieve a proper balance between too much and too little information in informed consent. Once the format and content of informed consent materials is done, translation and literacy issues need to be addressed. The informed consent form, mostly written in English first, is translated almost verbatim into the local language, thus complicating the explanation of concepts even more. The utility of good back-translation procedures has been reiterated as a possible solution for this. Another pragmatic approach, fostered by several studies over the last decade, is to involve the prospective subject’s community representatives in the process of developing the ICD. Involvement of community representatives ensures that consent form is structured and worded in the most appropriate possible manner to reach the understanding levels of the target population.[12] Community leaders and representatives may also be invited to the informational meetings for introducing the proposed research,[9,13] this helps to introduce them to the study and seek their reviews and comments that may help in designing ICD and in implementing the study later on. This is especially useful in scenarios where the social and cultural settings and sentiments of the target population are vastly different from that of the researchers. With more and more trials being conducted in developing countries for sponsors from developed countries, this holds much relevance. The crux of all efforts toward rendering the informed consent process with more authenticity is to reach the subject’s understanding and foster in them the sense of feeling adequately informed. A recent study suggests the use of computer-based approach to communicate about complex technical issues using visual aids, diagrams, videos, hyperlinks and quiz, to improve subject’s understanding of the study[14] Other studies have also emphasized the utility of audio-visual aids in enhancing the quality of information conveyed and retention of the knowledge by subjects.[15] The content of such audio visuals and other innovative interventions must be assessed to suit the understanding and education levels and cultural norms of people. Community outreach may also be improved by making use of illustrated booklets, videos, and drama skits, for use with illiterate populations.[16] Such decision aids help in increasing people’s involvement and is more likely to lead to informed decisions. Such aids if presented to a group of participants from a community, give them an opportunity to share their ideas and doubts actively; dialogue and discussions with peer group usually enhances understanding and eases off any unsettling feelings. It is a good way to boost them to drop their inhibitions and come out with all queries. Use of such aids has been shown to improve subject decision attributes, accurate risk perception, value congruence with chosen option and an overall high sense of feeling informed.[16]

Further after delivering all information, to complete the cycle of communication, it is important to assess how far the message has reached the target population. Researchers may ask the perspective participants to demonstrate their understanding of the information conveyed to them.[17] Such practice will also enable researchers to identify areas that are generally poorly grasped by the subjects. So they can be more illustrative about those elements and explain them carefully to the next participants.

It is very essential to have well-trained, knowledgeable staff to administer a fully comprehensible consent form in a private and comfortable setting that facilitates questioning from the potential participant. Generally patients who walk into a physician’s facility expect an examination and prescribed medication. To them, any deviation from this routine process may be a little unsettling. Hence, the participants must be allowed adequate time for discussions and decision making; and should be encouraged to ask questions without inhibitions. Research staff must be trained on human research participant protection and show adequate respect and patience during the process. A good tool to be used by research staff may be a list of the most often asked questions and adequate responses to them, that they may use to counsel and consent future participants.[18] Informed consent process combined with enhanced education and counseling materials can help in enhancing comprehension of the issues.[19]

It is important to remember that all forms of patient information aids need to be approved by ethics committee. International ethics guidelines are not clear about the potential array of materials that should be considered part of the informed consent process and reviewed by ethics committees. However, videos, skit, booklets and all other documents intended to be shared with the prospective research participants must be reviewed for content as well as appropriateness for target groups.[18]

A holistic approach to integrate apposite methods of information relay and ensure a truly autonomous informed consent; bringing together simplified consent document, illustrated patient decision aids, thorough counseling and education, to strengthen subject comprehension and credibility of a voluntary decision, needs to be fostered. A revision of the guidelines and regulations to mandate appropriate methods of gaining an informed, autonomous consent may be considered.[20] The emphasis needs to be shifted from laborious documentation of mechanical aspects of research process to assuring true comprehension and voluntary participation. The efforts are worth the time and cost involved, as it can strengthen the research effort through recruitment and retention of participants who better understand their roles and responsibilities in the study and can thus better adhere to the study protocol.

India has emerged as a clinical trial hub and the industry is spreading its tentacles fast over the population. Considering the paucity in medical awareness and exposure of our population, is it not imperative that the present guidelines and regulations regarding the informed consent be revisited? So that the true essence of Belmont Report, of *Respect, Beneficence and Justice, be fulfilled*!
